# Sanitary Clothing

**Published:** 1896-09

**Authors:** 


### SANITARY CLOTHING.

The materials of which underclothing is made are principally wool and silk from the animal, and cotton and linen from the vegetable kingdom. A great deal of ignorance prevails concerning the relative advantages of these, although this forms a question of very great importance, especially in relation to children and old people.

*Cotton* forms non-shrinkable, smooth, and durable garments, and being comparatively non-absorbent and a good conductor of heat, rapidly becomes wet and gives off the warmth of the body, causing a chill to its surface. The new "cellular" cotton cloth is made with such space between the fibers as allows of the presence of an abundance of non-conducting air, and is, therefore, less to be condemned than the ordinary material.

*Linen* resembles cotton, and is even inferior as a cloth for underwear.

*Wool* is a more expensive material than cotton, does not wear so well, and especially when washed in too hot water and with a soap containing much soda, and when wrung out before drying, garments made of this fiber are apt to harden and shrink. Some persons object to wool next the skin as they find it irritating. But in spite of these disadvantages it makes distinctly the best underwear for both cold and warm climates. The hygroscopic power of wool, or its capability of absorbing water into its substance, is double that of cotton in proportion to weight, and quadruple in proportion to surface, while it possesses also great water-holding power between its fibers—"water of interposition." It is also a very bad conductor of heat on account of the air contained in its interstices, and because of the presence in it of an animal oil, which in new woolen fiber may compose about fifteen per cent. of its substance.

It is not during exertion, but after it, that the danger of chilling occurs. Then the skin continues to give off watery vapor which, in the case of cotton clothing, passes to the outer surface and is there evaporated, at the same time, of course, lowering the temperature. In wool, on the other hand, the vapor is condensed, during which process the heat which had been rendered latent when the perspiration was vaporized, is given back, while the wool absorbs and retains the water, remaining therefore warm and dry, and preventing chilling. In hot climates, where chilling is a frequent precursor of ague, dysentery, and diarrhea, it is quite as important that wool should be worn next the skin as it is in cold countries in which the outer garments are usually of woolen material. It is believed in some places that wool worn next the skin diminishes the risk of malaria. Malarial poison is supposed to enter the system through the lungs or stomach, and clothing can hardly interfere with that, but that is no reason why clothing should not influence the susceptibility of individuals to the poisonous influences, and practical experience in West Africa, in Rome, and in other places seems to confirm the correctness of this view.

*Silk* in some respects resembles wool. It is a bad conductor of heat, but it is less absorbent than wool. It is cleanly, does not shrink much, and is pleasant to the skin. It comes next to wool as a material for underwear, but is very expensive, and not very durable. A mixture of wool and silk makes comfortable and useful underwear. For exposure to cold winds and rain *leather* and *gutta-percha* are invaluable, the objection to them being the prevention of the circulation of the air (wherein of course also lies their advantage) and the resulting discomfort when the wearer perspires. This can to some extent be obviated by proper ventilation.

For protection against the direct rays of the sun *color* is the point of importance. White is the best, then follow in order grey, yellow, pink, blue, and black. In the shade one color is as good as another.

Proper clothing is specially important for children and old people. The circulation of the latter is apt to be feeble, and heat regulation, like other functions, to be inefficient. Loss of heat cannot therefore be made good with the rapidity necessary for perfect health, and consequently the vital forces may be dangerously depressed.

In children the circulation is more rapid than in adults, and the blood in a given time passes more frequently to the surface of the body, where it loses heat; but more important is the fact that the superficial area of a body of the human shape is much greater in the child in proportion to its total bulk than in the grown person. In a cube with sides of one square foot the relation of surface to bulk is six to one, while in a cube with sides of two square feet the relationship is four to one. The same is true for cylinders, which more nearly resemble the human body. The special liability of children to lose heat is therefore evident, and it follows that in them it is of particular importance that the body should be wisely clothed.

